# Estimation of Prevalence of Thrombocytopenia in Cyanotic Congenital Heart Disease: A Cross-Sectional Study Among the Pediatric Population

**DOI:** 10.7759/cureus.55453

**Published:** 2024-03-03

**Authors:** Naman Mishra, Keta Vagha, Shraddha Sawhney, Siddhartha Murhekar, Jayant D Vagha, Chaitanya Kumar Javvaji

**Affiliations:** 1 Pediatrics, Jawaharlal Nehru Medical College, Datta Meghe Institute of Higher Education and Research, Wardha, IND; 2 Trauma and Orthopedics, Kings College Hospital NHS Foundation Trust, London, GBR

**Keywords:** tapvc, congenital heart defect, prevalence, cyanotic heart defects, tetralogy of fallot, thrombocytopenia

## Abstract

Background

Congenital heart disease (CHD) is one of the leading causes of mortality in India, with the majority being attributed to cyanotic conditions. Hence, it is crucial to assess the factors that play a significant role in patient prognosis in heart defects of a child. The present cross-sectional study assessed the prevalence of thrombocytopenia in patients with cyanotic congenital heart defects (CCHD). The objectives of our study were to assess the levels of platelets in various cyanotic congenital heart defects and then infer the prevalence of thrombocytopenia in these patients as a whole.

Methodology

The study population comprised children aged fifteen days to twelve years with two-dimensional (2D) echocardiography confirmation of CHD; those who were critically ill, had proven sepsis, and were not willing to participate in the study were excluded. Blood samples of enrolled patients were obtained and collected in ethylenediamine tetraacetic acid (EDTA) tubes for assessment. The prevalence was then calculated. Results were obtained and interpreted based on these observations.

Result

Out of 268 children with CHD, 52 reported thrombocytopenia, and the prevalence rate was found to be 19.4. The comparative analysis of thrombocytopenia showed a significant p-value only in cases with total anomalous pulmonary venous connection (TAPVC).

Conclusion

Patients with cyanotic congenital heart defects are often diagnosed with various hematological derangements, and while hemoglobin levels are usually seen to rise, significant thrombocytopenia is reported in these patients. The low platelet counts often pose a risk peri-surgically and can also affect the surgical outcomes of the patient. Therefore, it is imperative to study further the relationship between thrombocytopenia and an independent risk factor for patient prognosis in patients of CCHD.

## Introduction

Congenital heart disease (CHD) is one of the most common developmental anomalies in children. With a mean incidence of 1.4 per 1000 live births worldwide and 200,000 per year in India, cyanotic congenital heart diseases (CCHD) significantly cause morbidity in the pediatric population [1,2]. CCHDs represent diverse medical conditions with varying pathophysiological mechanisms influencing their clinical manifestations. Among these conditions, certain anomalies like transposition of the great arteries (TGA) and total anomalous pulmonary venous connection (TAPVC) typically manifest during the neonatal period. Conversely, tetralogy of Fallot (TOF) and related disorders typically exhibit symptoms such as cyanotic spells and a silent chest without apparent signs of congestive heart failure, which get relieved on squatting. In contrast, the physiology associated with transposition heart defects tends to present with congestive heart failure accompanied by cyanosis [3]. 

Cyanotic cardiac diseases often affect multiple systems and present with various hematological abnormalities [4]. The conditions often include anemia, coagulation abnormalities, ischemia hyperviscosity, and thrombocytopenia. Thrombocytopenia, characterized by a low platelet count, is a common hematologic abnormality observed in individuals with CHDs. Various theories have been proposed to account for thrombocytopenia in individuals with CCHD, yet none have been substantiated. Among these theories, one postulates that prolonged hypoxia triggers complex alterations in whole blood composition and coagulation profiles. In this context, thrombocytopenia is a notable risk factor in CCHD patients. In such cases, the exacerbation of right-to-left shunting, resulting in increased pulmonary circulation bypass, may impede the typical fragmentation of megakaryocytes within the lungs [4]. Thrombocytopenia is occasionally severe in cases of cyanotic heart diseases, and the patients are operated on despite the low platelet counts, which improve after the corrective surgery [5].

This research aims to shed light on the prevalence of thrombocytopenia in individuals with congenital heart defects. By analyzing patients based on their complete blood count reports, this study provides a quantitative estimate of the prevalence of thrombocytopenia in this population. The findings will help healthcare professionals improve the management and care of patients with CHDs by raising awareness about the frequency and significance of thrombocytopenia.

## Materials and methods

Study design and area

This cross-sectional study was conducted by researchers from the Department of Pediatrics, Acharya Vinoba Bhave Rural Hospital, Sawangi (Meghe), in Maharashtra, India. The patients were recruited from January 2023 to December 2023 after obtaining due ethical approval from the Institutional Ethical Committee. The patients in the study were recruited from the hospital's pediatrics and cardiology outpatient departments. 

Study population

Purposive sampling was done to recruit the study participants. A total of 280 patients with CCHD were selected. The sample size had a confidence interval of 95% with a 5% margin of error. The average number of patients reporting to the pediatric outpatient department (OPD) yearly with congenital heart defects is 1100, of which the population proportion of cyanotic congenital heart defects is 60%, making the sample size 277-280, calculated using the Cochran formula.

Inclusion and exclusion criteria

Children with cyanotic congenital heart defects that were confirmed on echocardiography were included in the study. Children whose parents did not consent to their study enrollment, children with sepsis features evident by positive history or lab investigations suggestive of leucocytosis/leukopenia, elevated C-reactive protein (CRP), or positive blood culture and children diagnosed with congenital syndromes like Downs, Edward and Noonan were excluded from the study.

Methodology

**Figure 1:**
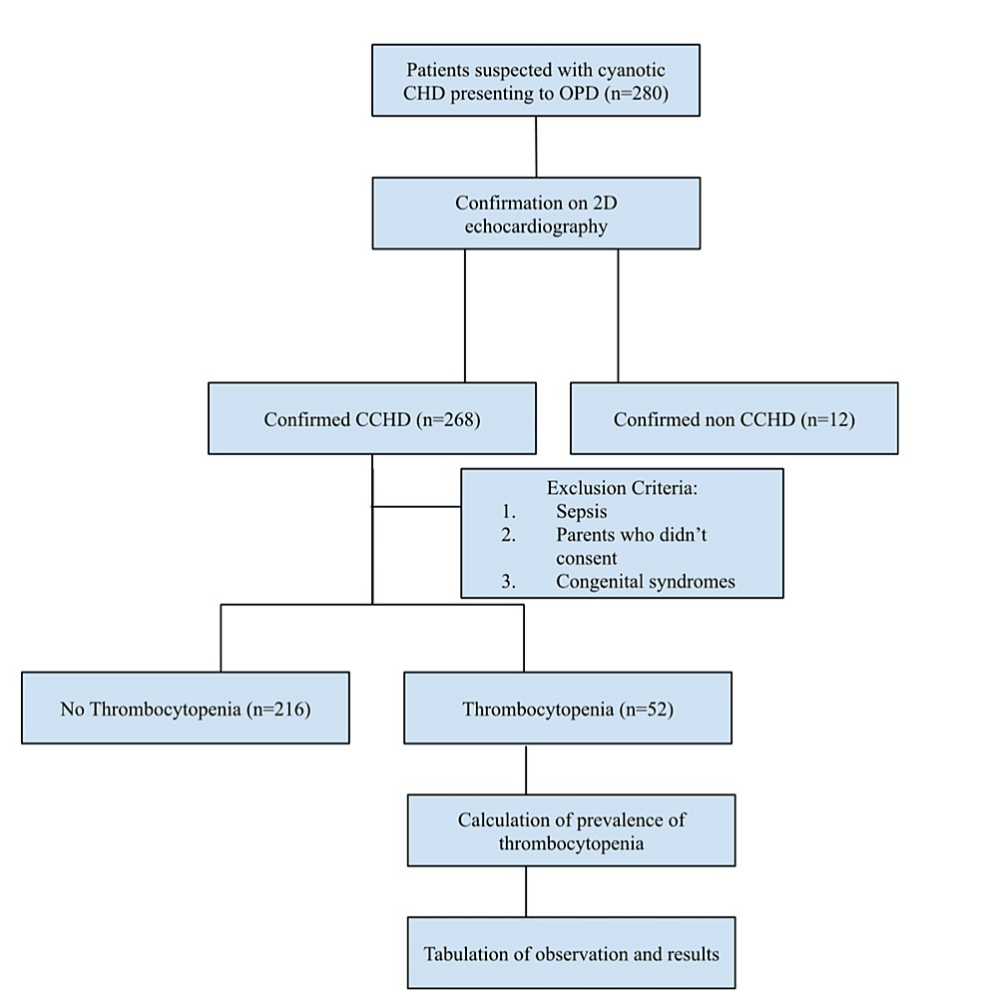
Methodology of the research CHD - congenital heart defects; CCHD - cyanotic congenital heart defects

The study population consisted of children who reported to the outpatient department of pediatrics or cardiology with symptomatology consistent with cardiac issues, underwent 2D echocardiography, and were diagnosed with CCHD like tetralogy of Fallot, truncus arteriosus, transposition of great arterioles, Ebstein anomaly and total anomalous pulmonary venous connection. Children with features of sepsis, evident by positive history and lab investigations suggestive of leucocytosis/leukopenia, elevated CRP, and positive blood cultures, were excluded from the study. The children were examined, and their systemic examination findings were noted along with documentation of vitals like heart rate, respiratory rate, blood pressure, and four limb oxygen saturation by pulse oximetry. Blood samples were taken for a detailed investigation of the platelet counts and hemoglobin estimations after getting informed written consent from their parents. Two milliliters (2 ml) of blood were taken aseptically from the study participants via venepuncture and administered into the ethylenediamine tetraacetic acid (EDTA) bulb. A trained phlebotomist collected the blood samples and labeled them appropriately. After the samples arrived at the laboratory, the platelet counts and hemoglobin levels were determined using the Beckman Coulter DxH 900 (Beckman Coulter, Inc., Brea, US) hematology analyzer. A count of less than 150,000 was termed thrombocytopenic. A good rapport and confidentiality were maintained while assessing the values. Based on these reports, observations and results were formulated. The methodology is depicted in the flow chart, as seen in Figure 1.

Statistical analysis

A chi-square or Fisher's exact test was used to compare categorical data between the groups. The data was analyzed using the SPSS (version 25.0, IBM Inc., Armonk, US) and Microsoft Excel 2016 (Microsoft Corp., Redmond, US). The level of statistical significance was achieved if p<0.05. Demographic data was collected for the study through the researchers' registration of the patients in the OPD. The mean score was calculated and rated. 

Ethical considerations

The study was conducted after approval from the institutional ethical committee, and permission from the hospital was obtained to conduct the research. Written and verbal consent was obtained from the patients under study, and they were informed about the objectives and implications of the research. The participants were ensured privacy and confidentiality. The study protocol was reviewed and approved by the DMIMS (DU)/IEC/Dec-2022/1216.

## Results

Two hundred and eighty patients were enrolled in the study, of which 268 were confirmed to have cyanotic congenital heart defects on 2D echocardiography. The mean age of the subjects was eight months, with a minimum age of 15 days and a maximum age of 12 years. One hundred and twenty-six (47.01%) were males, and 142 (52.98%) were females (Table 1).

**Table 1:** Demographic details of the sample

Characteristics	Subcategories	Number	Percentage
Age	Below one year	240	89.56
	1-3 years	16	5.98
	4-6 years	6	2.23
	6-12 years	6	2.23
Gender	Male	126	47.02
	Female	142	52.98

**Figure 2:**
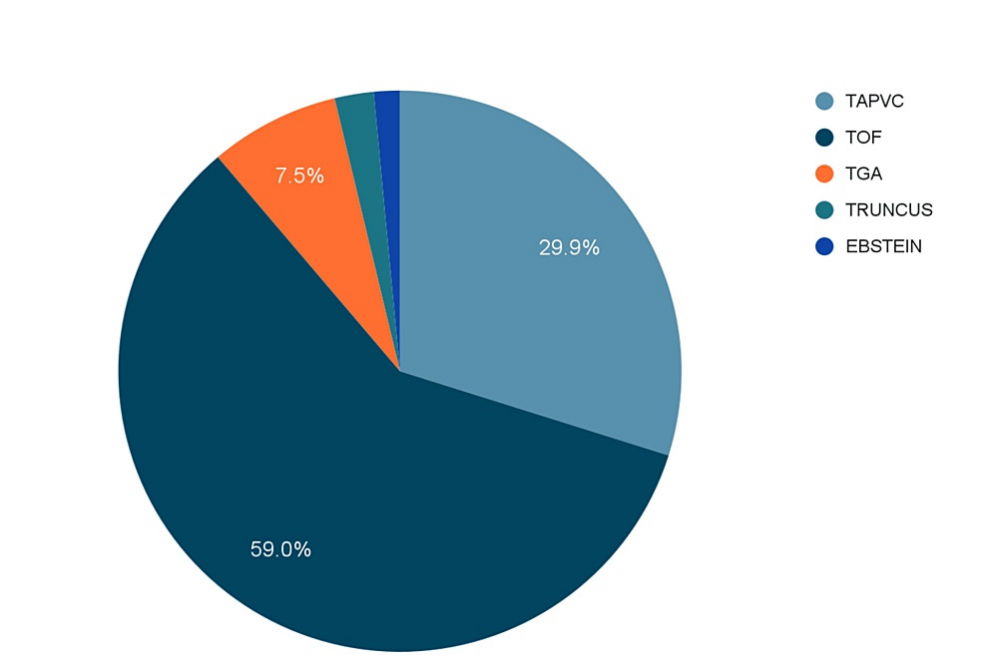
Type of cyanotic heart defect TAPVC - total anomalous pulmonary venous connection; TOF - tetralogy of Fallot; TGA - transposition of great arteries; Truncus - truncus arteriosus; Ebstein - Ebstein anomaly

Out of the 268 children, a variety of cyanotic heart diseases were observed, namely total anomalous pulmonary venous connection (TAPVC) in 29.9%, truncus arteriosus in 2.2%, tetralogy of Fallot (TOF) in 59%, transposition of great arterioles (TGA) in 7.5% and Ebstein anomaly in 1.5% (Figure 2).

While most patients in the study population were diagnosed with TOF, TGA corresponded to the maximum number of thrombocytopenics. With a total of 20 children suffering from the disease, six (30%) had thrombocytopenia. Out of the total patients with cyanotic heart disease, 52 showed the presence of thrombocytopenia. The number of cases of thrombocytopenia in the male population (n=28) was slightly higher than that of the female patients (n=24). On comparing the specific cyanotic disease among thrombocytopenic patients, patients with TGA had the maximum association with thrombocytopenia, followed by TOF and TAPVC. However, other congenital diseases like Ebstein anomaly and truncus arteriosus did not show the presence of thrombocytopenia, which could also be attributed to the smaller number of these cases reporting to the OPD. The relationship of thrombocytopenia in cases of TAPVC showed a significant p-value of 0.002 compared to the non-thrombocytic TAPVC cases. The hemoglobin concentration among those suffering from thrombocytopenia was 15.65±3.96, while the patients with no thrombocytopenia had an Hb concentration of 13.87±3.18. This result shows a significant increase in Hb concentration in the patients who suffer from thrombocytopenia. The oxygen saturation also decreased in patients with thrombocytopenia (Table 2).

**Table 2:** Characteristics of patients with and without thrombocytopenia TAPVC - total anomalous pulmonary venous connection; TOF - tetralogy of Fallot; TGA - transposition of great arteries; Hb% - hemoglobin percentage; ns - non-significant; s - significant

Variable	Thrombocytopenia	No thrombocytopenia	p-value
Female	24	104	0.87, ns
Male	28	112	0.87, ns
TAPVC	8	72	0.002, s
TGA	6	14	0.60, ns
Ebstein anomaly	0	4	1.00, ns
TOF	38	120	1.00, ns
Truncus arteriosus	0	6	0.60, ns
Hb%	15.65±3.96	13.87±3.18	0.001, s
O_2_ saturation	79.00±5.46	84.9±6.5	0.57, ns
Platelet count	1.03±0.34	2.48±0.86	0.0001, s

The prevalence of thrombocytopenia in the study population is 19.4%, which shows an association between thrombocytopenia and congenital cyanotic heart disease. The prevalence was calculated as follows:

$$Prevalence=\frac{no.\;thrombocytopenia\;patients}{total\;study\;population}*100\frac{52}{268}*100=19.4$$

## Discussion

**Figure 3:**
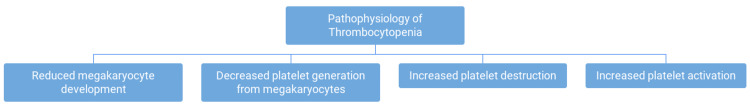
Postulated pathophysiology of thrombocytopenia in CCHD CCHD - cyanotic congenital heart diseases

Thrombocytopenia has a strong association with cyanotic heart diseases and, therefore, can pose a significant risk in patients undergoing cardiopulmonary bypass or shunt surgeries [5]. Therefore, it is imperative to study the association between these two conditions. This study demonstrates a significant relationship between thrombocytopenia and CCHD. By analyzing data on changes in hematological parameters, like a significant difference between the hemoglobin and platelet count values between patients with thrombocytopenia and non-thrombocytopenia and varied oxygen saturation levels, our study adds to understanding thrombocytopenia in cyanotic CHD. Though the exact causation of thrombocytopenia in CHD patients is not known, it can occur through four different pathways. There can be reduced megakaryocyte development, decreased platelet generation from megakaryocytes, increased platelet destruction, or increased platelet activation (Figure 3).

The underlying pathophysiology is associated with right-to-left shunts, which redirect platelet precursors from systemic venous to arterial circulation, bypassing the lungs and reducing platelet formation in the pulmonary bed. Megakaryocytic cells traverse these shunts and release platelets at systemic impact locations; nevertheless, these platelets remain localized and do not contribute to total circulation platelet numbers [6]. Polycythemia, higher blood viscosity, and other rheological variables all enhance the release of adenine diphosphate, which can lead to thrombocyte aggregation and thrombocytopenia [7]. A reduced lifespan of the platelets can also be attributed to an increased thrombocytolysis or reduced number of platelets synthesized [8]. 

Cui et al. stated that changes in megakaryocyte metabolism contributed significantly to the low platelet counts reported in cyanotic patients [9]. This proposition gave rise to the notion that the severity of thrombocytopenia is primarily determined by the extent of underlying pathology, as evidenced by the degree of right-to-left shunting resulting in hypoxemia and cyanosis. This might explain why thrombocytopenia has such a significant predictive value. 

The study had 268 participants, of which 126 were males and 142 females, and the male-to-female ratio was 1:1.1. This is contrary to the ratio in the studies done in Pakistan, which were 1.31:1 and 1.32:1, respectively [10,11]. This study showed that 19.4% of the total patient population of CCHD had thrombocytopenia. This was inconsistent with the study done by Chamanian et al. in the adult population, where thrombocytopenia was found in 30% of patients [12]. This difference could be attributed to the fact that the cut-off platelet levels used to determine thrombocytopenia were different in both studies. 

The present study found that the mean of the platelets was inversely proportional to the mean of the hemoglobin values in the thrombocytopenic and non-thrombocytopenic groups, which is comparable to the findings of the studies conducted by Danioth et al., where a substantial correlation was seen between changes in platelet counts and inverse changes in hemoglobin levels [4] and Gross et al., who carried out his study on cyanotic heart disease patients in the neonatal age group [13]. The study by Horigome et al. also corroborated the association of polycythemia with thrombocytopenia in patients with CCHD [14].

In our study, the most common cyanotic heart disease was TOF, and the least common was Ebstein anomaly, comparable to the findings of a hematological study by Animasahun et al. [15].

Thrombocytopenia was found to be major in patients diagnosed with TGA, which was 30% of the patients suffering from the transposition of great arterioles. A study showed that the maximum number of patients with thrombocytopenia were diagnosed with Eisenmenger syndrome [4]. One possible explanation for this discrepancy could be the difference in the age group of the study population. Although thrombocytopenia is not the direct cause of mortality in patients with CCHD, more research and analysis are needed to fully explore its potential as a significant independent risk marker. 

Limitations

The limitations of the study are the small sample size, the lack of carrying out thromboelastography due to limited resources, and the lack of assessment of the correlation between other hematological derailments.

## Conclusions

This study focused on assessing the prevalence of thrombocytopenia in patients with congenital cyanotic heart diseases. The study concludes a significant association between thrombocytopenia and congenital cyanotic heart diseases. Hence, managing the platelet count becomes imperative before undergoing shunt or bypass surgeries. Fifty-two of the 268 patients tested positive for thrombocytopenia (platelet count <1.5 lakh). The prevalence in the study was 19.4%. Furthermore, our findings indicate a direct inverse relationship between the mean platelet count and mean hemoglobin concentration levels in individuals with congenital cyanotic cardiac disorders. To determine their impact on patient prognosis, further research is needed to assess platelet count and other hematological derangements like coagulopathies.
